# Supplementary material for: Intracellular lipid droplet accumulation occurs early following viral infection and is required for an efficient interferon response
Source: Nat Commun. 2021 Jul 14;12:4303. doi: 10.1038/s41467-021-24632-5 (PMC8280141; doi:10.1038/s41467-021-24632-5)
Supplement: Supplementary file 1 — Supplementary information [file 41467_2021_24632_MOESM1_ESM.pdf]

Supplementary Information

Intracellular Lipid Droplet Accumulation Occurs Early Following Viral Infection and Is Required for an Efficient Interferon Response

EA Monson[1], KM Crosse[1], M Duan[2], W Chen[2], RD O'Shea[1], LM Wakim[3], JM Carr[4] DR Whelan[2], KJ Helbig[1]

[1] School of Life Sciences, La Trobe University, Melbourne, Australia;

[2] La Trobe Institute for Molecular Science, La Trobe University, Melbourne, Australia.

[3] Department of Microbiology and Immunology, University of Melbourne, at Peter Doherty Institute for Infection and Immunity, Melbourne, Australia.

[4] Microbiology and Infectious Diseases, College of Medicine and Public Health, Flinders University, Adelaide, Australia

Correspondence to be addressed to K.J.H (email k.helbig@latrobe.edu.au).

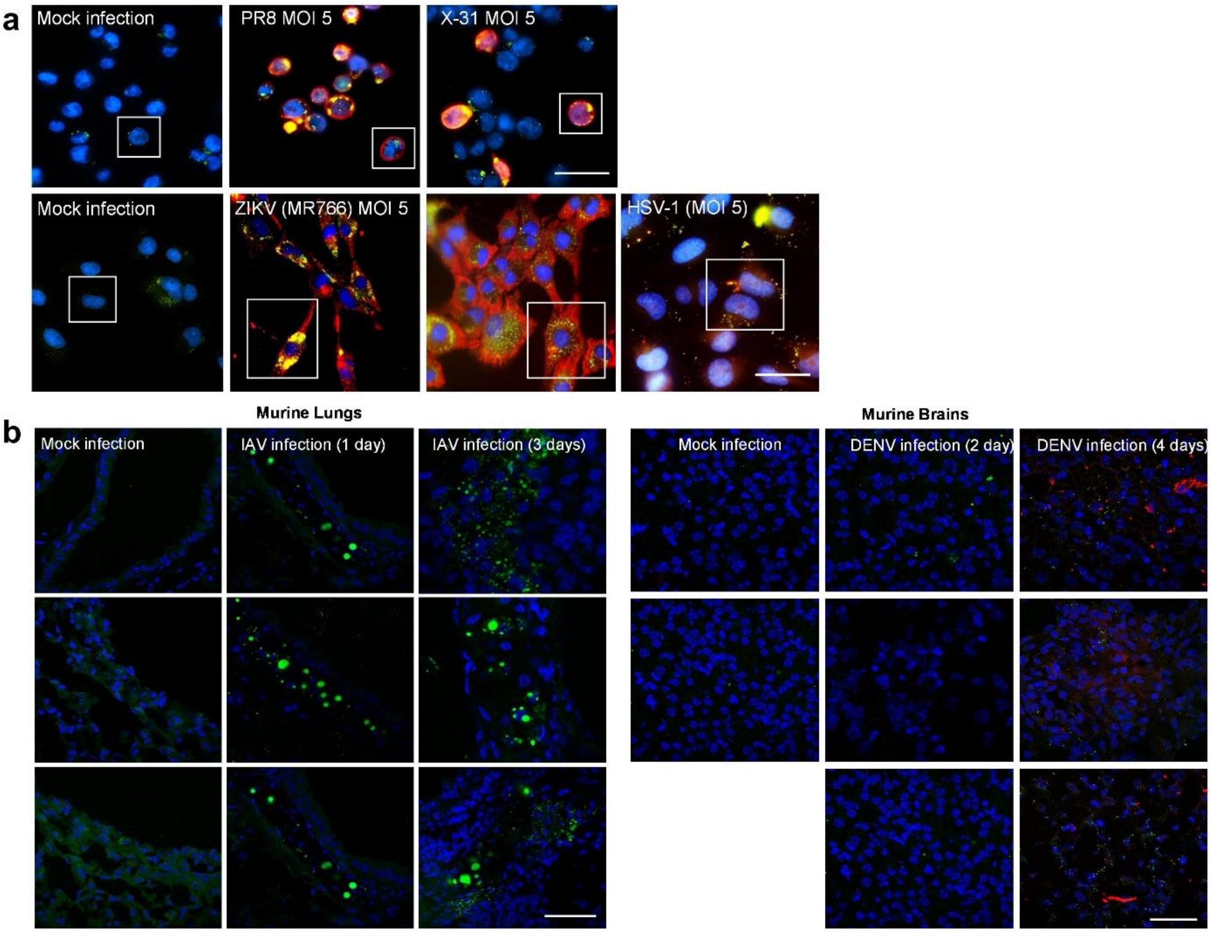

Supplementary Figure 1. Influenza, ZIKV and HSV-1 virus infection stimulated the induction of lipid droplets. (a) Human THP-1 monocytes were infected with two different strains of IAV; PR8 and X-31 at MOI 5. Primary immortalised astrocyte cells were infected with either the ZIKV (MR766 strain) or HSV-1 (KOS strain) at MOI 5. Cells were stained with Bodipy (493/503) to visualise LDs (green) and DAPI to visualise the cell nuclei (blue). IAV was detected with an αNS2 antibody, ZIKV RNA was detected using an anti-3G1.1 and 2G4 dsRNA antibodies and HSV-1 was detected using the anti-HSV-1 antibody (Abcam, ab9533) (all viral stains shown in red). White boxes indicate the zoomed images of Fig. 1A & Fig. 1B. Scale bars, 50μm. Images are a representation of n=3 independent experiments. **(b)** C57BL/6 mice were either mock infected or infected with influenza A virus for either 1 or 3 days prior to removal of both lung lobes for immunofluorescence analysis. 1-day old BALB/c pups were either mock infected or infected with 800 PFU of DENV-2 (MON601) for 2 or 4 days prior to removal of pup heads for immunofluorescence analysis. Images each represent n=3 replicate mice. Analysis of LDs was performed via Bodipy (493/503) staining (green) in the murine lungs, brains, and eyes. DAPI was utilised to visualise the cell nuclei (blue). DENV RNA was

detected using anti-3G1.1 and 2G4 dsRNA antibodies (in combination). Scale bars, 50μm for lung sections and 500μm for brain sections.

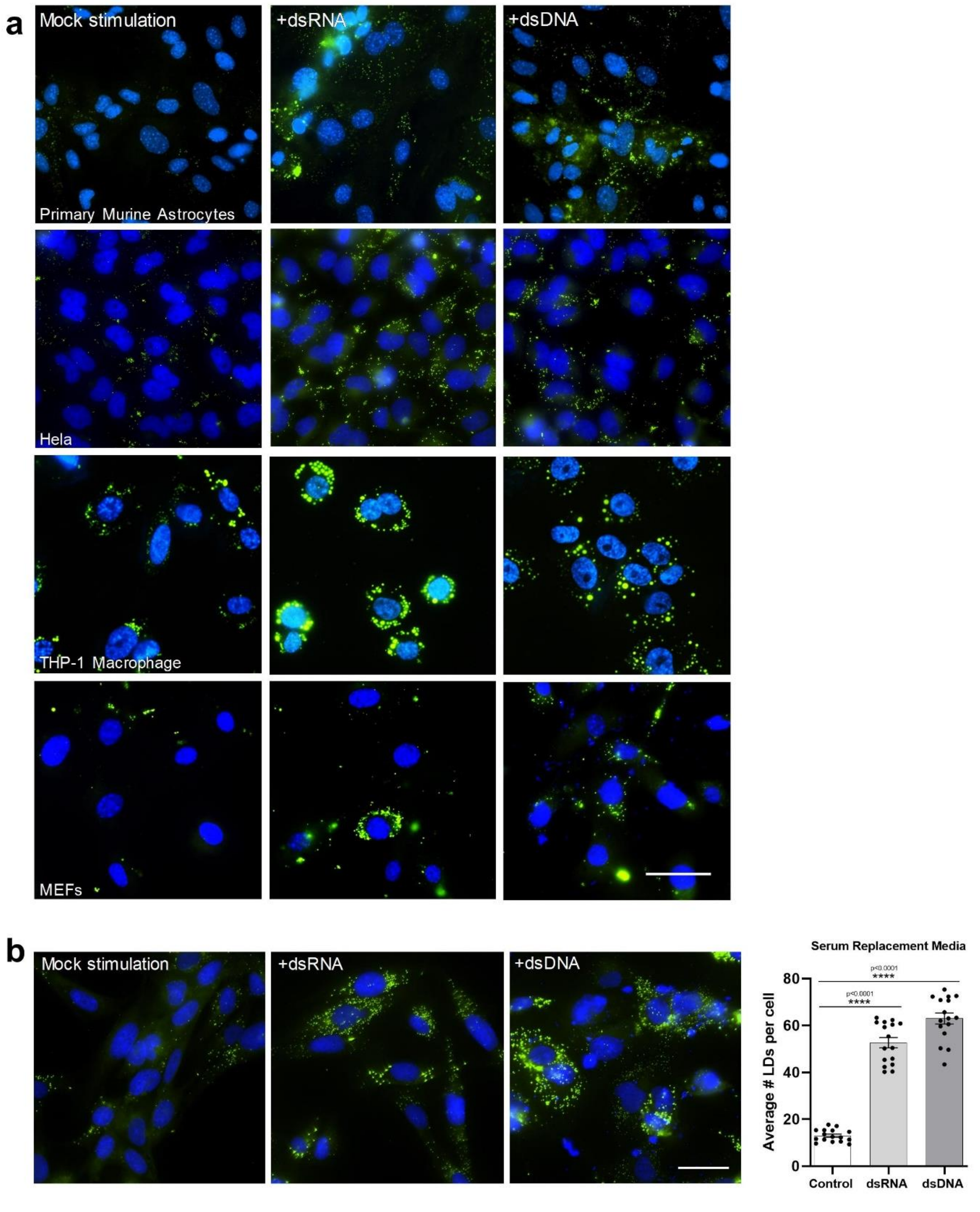

Supplementary Figure 2. Lipid droplets accumulate in multiple cell types in response to detection of dsRNA and dsDNA. **(a)** Primary murine astrocyte, HeLa, THP-1 macrophages and MEF cells were stimulated with dsRNA and dsDNA for 8hrs and stained with Bodipy (493/503) to visualise LDs (green) and DAPI to visualise the cell nuclei (blue). Cells were imaged on a Nikon T*i*E microscope. Original magnification is 60X. Images are a representation of n=3 independent experiments. **(b)** To assess if this induction was dependent on fetal bovine serum in the cell media primary immortalised astrocyte cells were grown in Serum

Replacement Media 3 (Sigma), seeded on coverslips and stimulated with dsRNA and dsDNA for 8 hrs. Cells were stained with Bodipy (493/503) to visualise LDs (green) and DAPI to visualise the cell nuclei (blue), and average number of LDs per cell analysed using ImageJ analysis software. Error bars, mean values ± SEM, P-values were determined by unpaired two-tailed Student's t test with a Holm-Sidak correction for multiple comparisons (greater than 300 cells; n=3 biological replicates). Stimulated cells were statistically compared with their respective mock controls. Scale bars, 50µm. Source data are provided as a Source Data file.

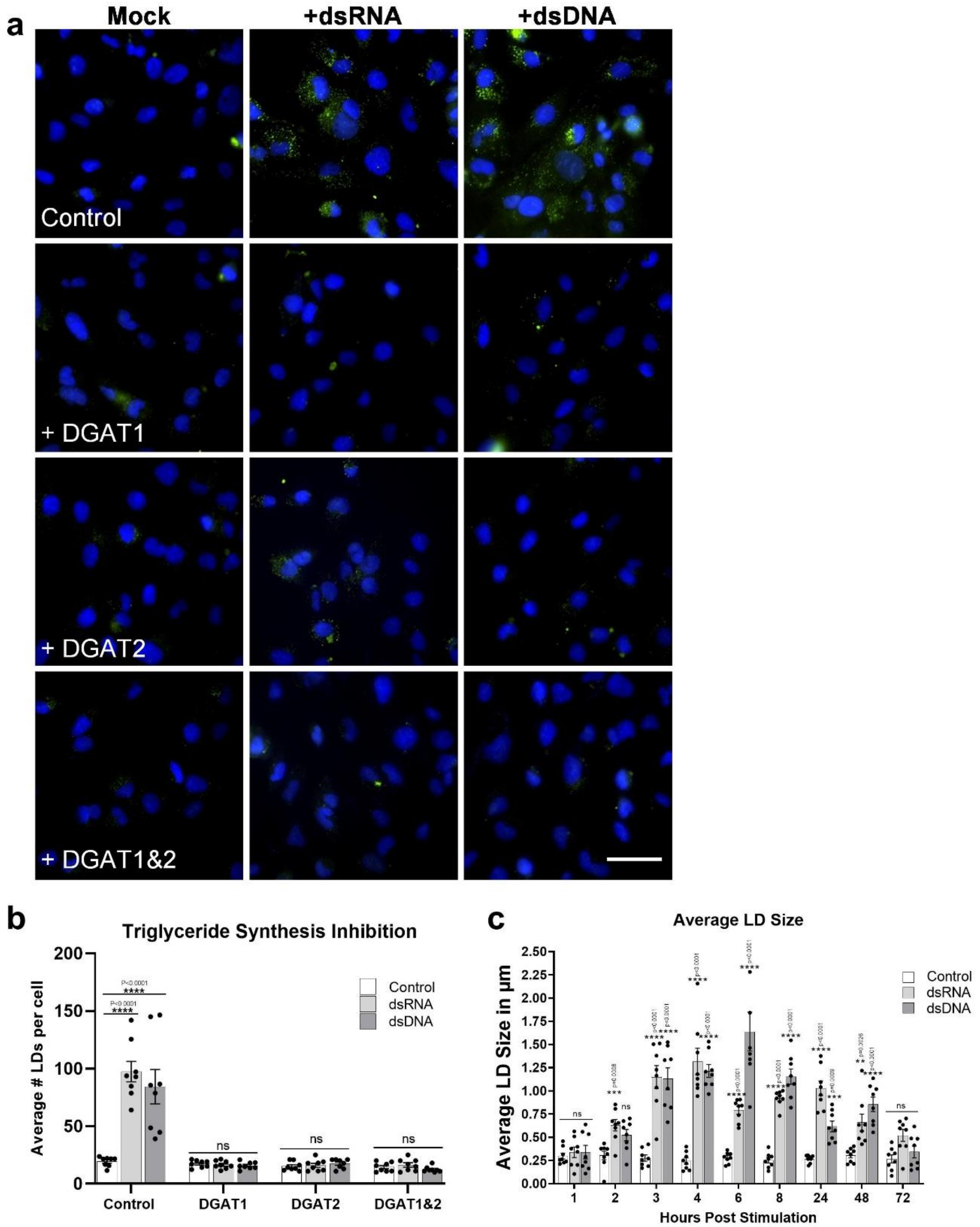

Supplementary Figure 3. The average size of lipid droplet increases following detection of dsRNA and dsDNA and returns to basal sizes at 72 hrs. (a) Primary immortalised astrocyte cells were treated with either the DGAT1 inhibitor, T863 or the DGAT2 inhibitor, PF-06424439 (or a combination of both) for 1 hr prior to cell stimulation. Cells were stimulated with dsRNA and dsDNA for 8 hrs. Cells were stained with Bodipy (493/503) to visualise LDs (green) and DAPI to visualise the cell nuclei (blue). Images are a representation of n=3

independent experiments. **(b)** Average LD size was analysed using ImageJ analysis software. **(c)** Primary immortalised astrocyte cells were stimulated with dsRNA and dsDNA and were fixed at regular time points up to 72 hrs. Average size (diameter) of LDs per cell were analysed from all time points using ImageJ analysis software. In **b & c** error bars, mean values ± SEM, P-values were determined by unpaired two-tailed Student's t test with a Holm-Sidak correction for multiple comparisons (greater than 300 cells; n=3 biological replicates). Stimulated cells were statistically compared with their respective mock controls, ns= not significant. Source data are provided as a Source Data file.

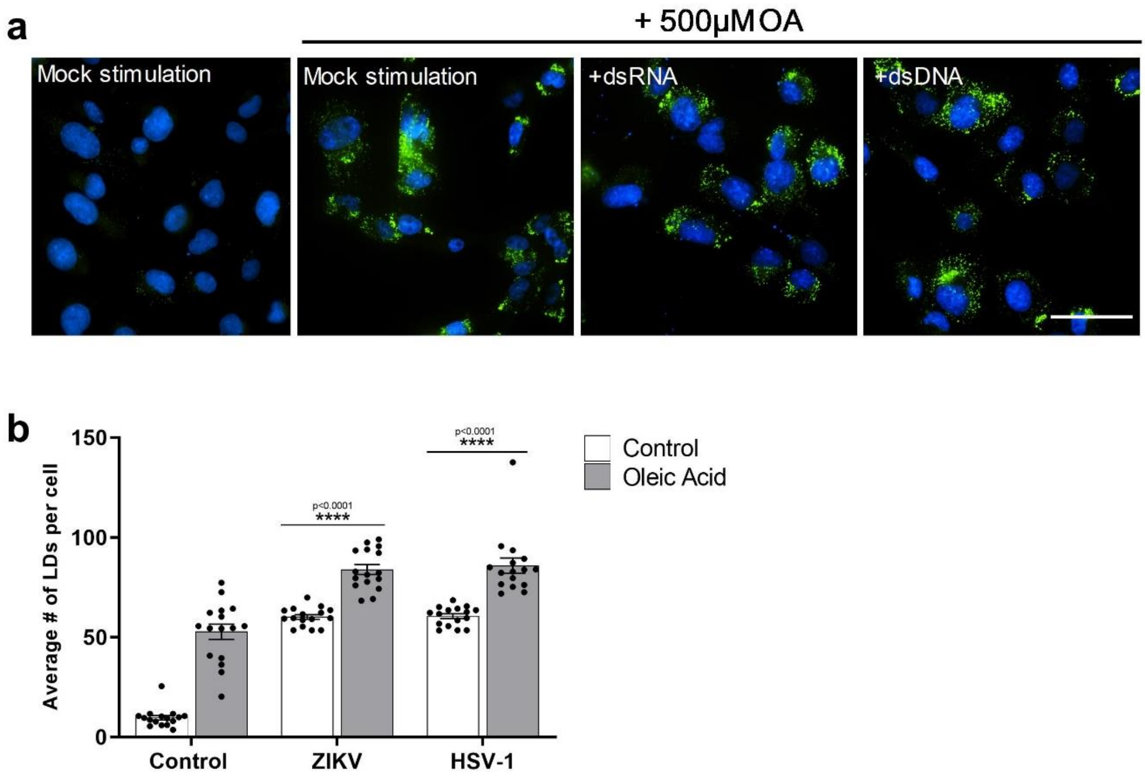

Supplementary Figure 4. Lipid droplets continue to accumulate following dsRNA and dsDNA after oleic acid treatment. (a) Primary immortalised astrocyte cells were treated with 500µM oleic acid (OA) for 16 hrs, prior to stimulation with dsDNA or dsRNA. Cells were stained with Bodipy (493/503) to visualise LDs (green) and DAPI to visualise the cell nuclei (blue). Bars, 50µm. Images are a representation of n=3 independent experiments. **(b)** Primary immortalised astrocyte cells were treated with 500µM OA for 16 hrs prior to infection with either ZIKV or HSV-1 (MOI 1) and the average number of LDs was analysed per cell with ImageJ analysis software. Error bars, mean values ± SEM, P-values were determined by unpaired two-tailed Student's t test with a Holm-Sidak correction for multiple comparisons (greater than 300 cells; n=3 biological replicates). Stimulated cells were statistically compared with their respective mock controls. Source data are provided as a Source Data file.

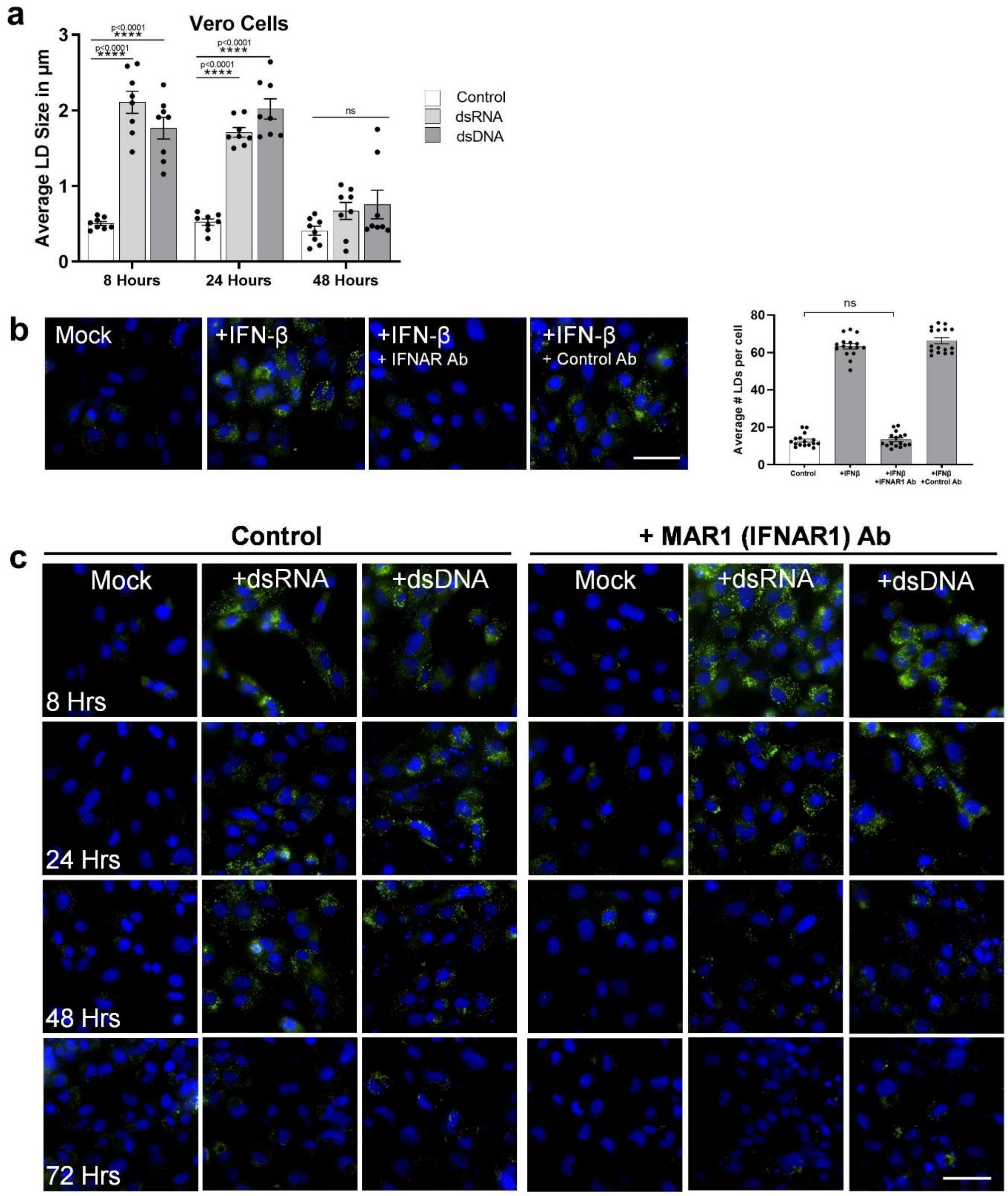

Supplementary Figure 5. The average size of lipid droplet increases following detection of dsRNA and dsDNA in Vero cells. (a) Vero cells were stimulated with dsRNA and dsDNA and were stained with Bodipy (493/503) to visualise LDs (green) and DAPI to visualise the cell nuclei (blue) at 8, 24 and 48 hrs post stimulation and LD sizes (diameters) were analysed using ImageJ analysis software. **(b)** Primary immortilised astrocyte cells were treated with MAR1 (anti IFNAR1) antibody to block type-I IFN signalling 1 hr prior to stimulation. Cells were then stimulated with IFN-β and cells were stained with Bodipy (493/503) to visualise LDs (green) and DAPI to visualise the cell nuclei (blue). Average LD size was analysed using

ImageJ analysis software. In **a & b** error bars, mean values ± SEM, P-values were determined by unpaired two-tailed Student's t test with a Holm-Sidak correction for multiple comparisons (greater than 300 cells; n=3 biological replicates). Stimulated cells were statistically compared with their respective mock controls **=p<0.01, ***=p<0.001, ns= not significant. **(c)** Primary immortilised astrocyte cells were treated with MAR1 1 hr prior to stimulation with dsRNA and dsDNA, and cells were stained with Bodipy (493/503) to visualise LDs (green) and DAPI to visualise the cell nuclei (blue). Scale bars, 50μm. Source data are provided as a Source Data file.

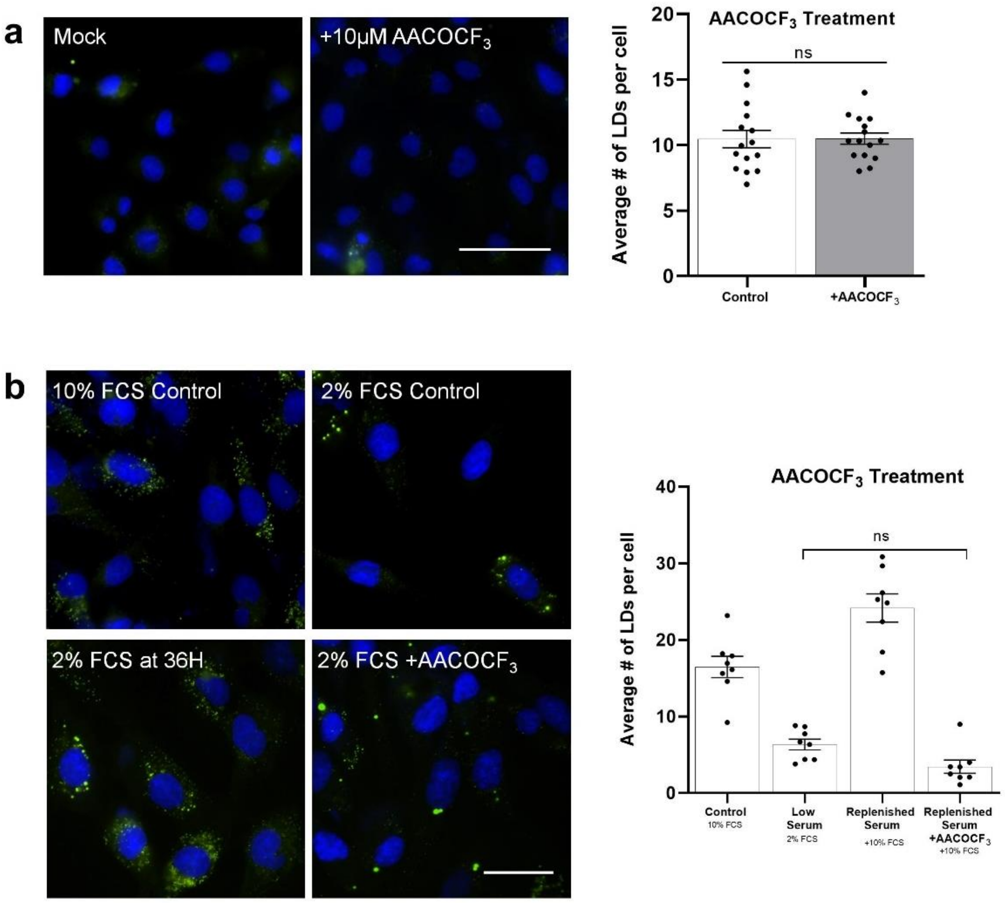

Supplementary Figure 6. AACOCF$_3$ treatment inhibits the homeostatic biogenesis of lipid droplets. (a) Primary immortalised astrocyte cells were treated with 2µM AACOCF$_3$ for 16 hrs and LD numbers were compared to control treated cells using ImageJ analysis software. **(b)** Primary immortalised astrocyte cells were serum starved for 48 hrs, plated into wells and treated with 2µM AACOCF$_3$ (PLA$_2$ inhibitor) or left as control cells for 16 hrs. All cells were then given fresh full serum media for 36 hrs and stained with Bodipy (493/503) to visualise LDs (green) and DAPI to visualise the cell nuclei (blue), and average number of LDs per cell analysed using ImageJ analysis software. Error bars, mean values ± SEM, P-values were determined by unpaired two-tailed Student's t test with a Holm-Sidak correction for multiple comparisons (greater than 300 cells; n=3 biological replicates). Treated cells were statistically compared with their respective mock controls, ns= not significant. Scale bars, 50µm. Source data are provided as a Source Data file.

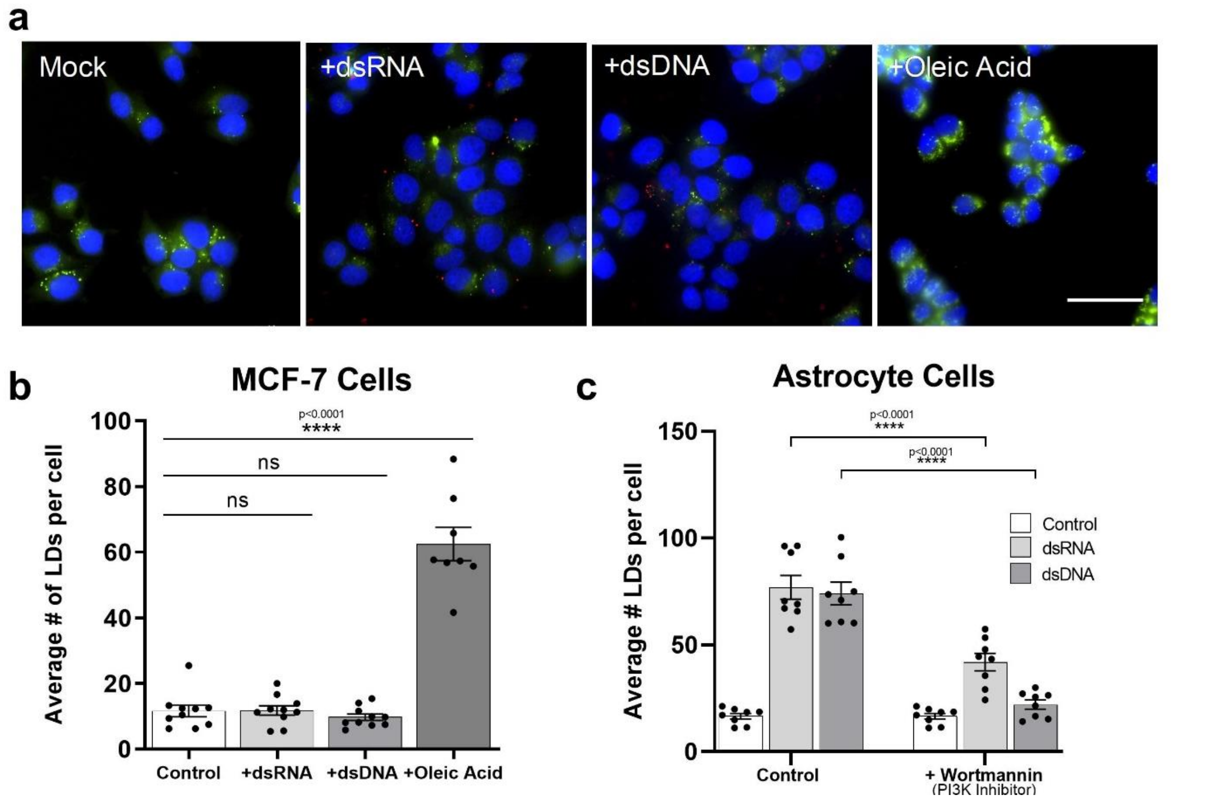

Supplementary Figure 7. EGFR and PI3K control the induction of virally induced LDs. **(a)** MCF-7 cells (known to lack EGFR) were stimulated with dsRNA and dsDNA for 8 hrs and visualised for LD numbers and **(b)** analysed using ImageJ analysis software. **(c)** Primary immortalised astrocyte cells were stimulated with Wortmannin (PI3K inhibitor) and stimulated with dsRNA and dsDNA and their LD numbers were analysed using ImageJ analysis software. Error bars, mean values ± SEM, P-values were determined by unpaired two-tailed Student's t test with a Holm-Sidak correction for multiple comparisons (greater than 200 cells; n=2 biological replicates). Stimulated cells were statistically compared with their respective mock controls, ns= not significant. Scale bars, 50μm. Source data are provided as a Source Data file.

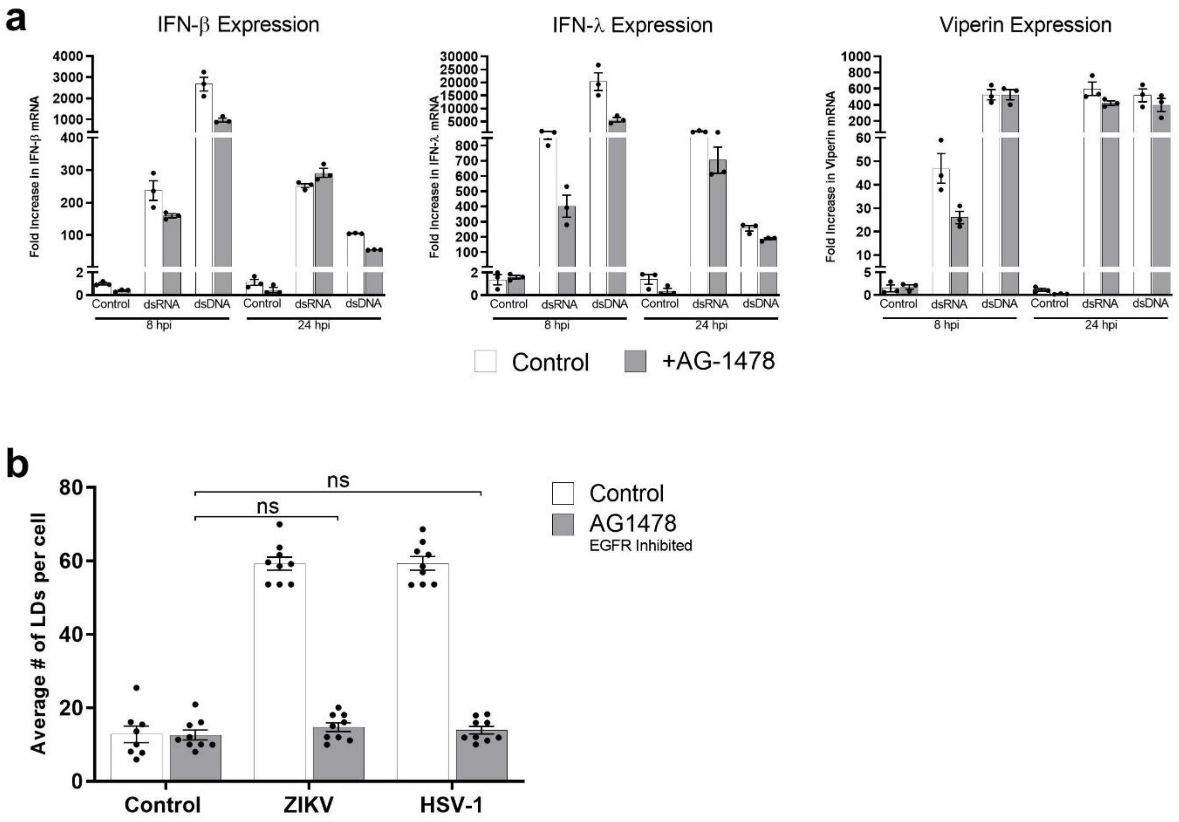

Supplementary Figure 8. AG-1478 treatment reduces type I and III IFN production in primary immortalised astrocyte cells following dsRNA and dsDNA stimulation. Primary immortalised astrocyte cells were treated with 2μM AG-1478 (EGFR inhibitor) for 16 hrs prior to stimulation with dsDNA or dsRNA and **(a)** RT-qPCR was performed to evaluate IFN-β, IFN-λ and viperin mRNA expression at 8 hrs and 24 hrs post stimulation. **(b)** Primary immortalised astrocyte cells were treated with 2μM AG-1478 (EGFR inhibitor) for 16 hrs prior to infection with either ZIKV or HSV-1 (MOI 1) and the average number of LDs was analysed per cell with ImageJ analysis software. In **a & b** error bars, mean values ± SEM, P-values were determined by unpaired two-tailed Student's t test with a Holm-Sidak correction for multiple comparisons (greater than 300 cells; n=3 biological replicates). Stimulated cells were statistically compared with their respective mock controls, ns= not significant. Source data are provided as a Source Data file.

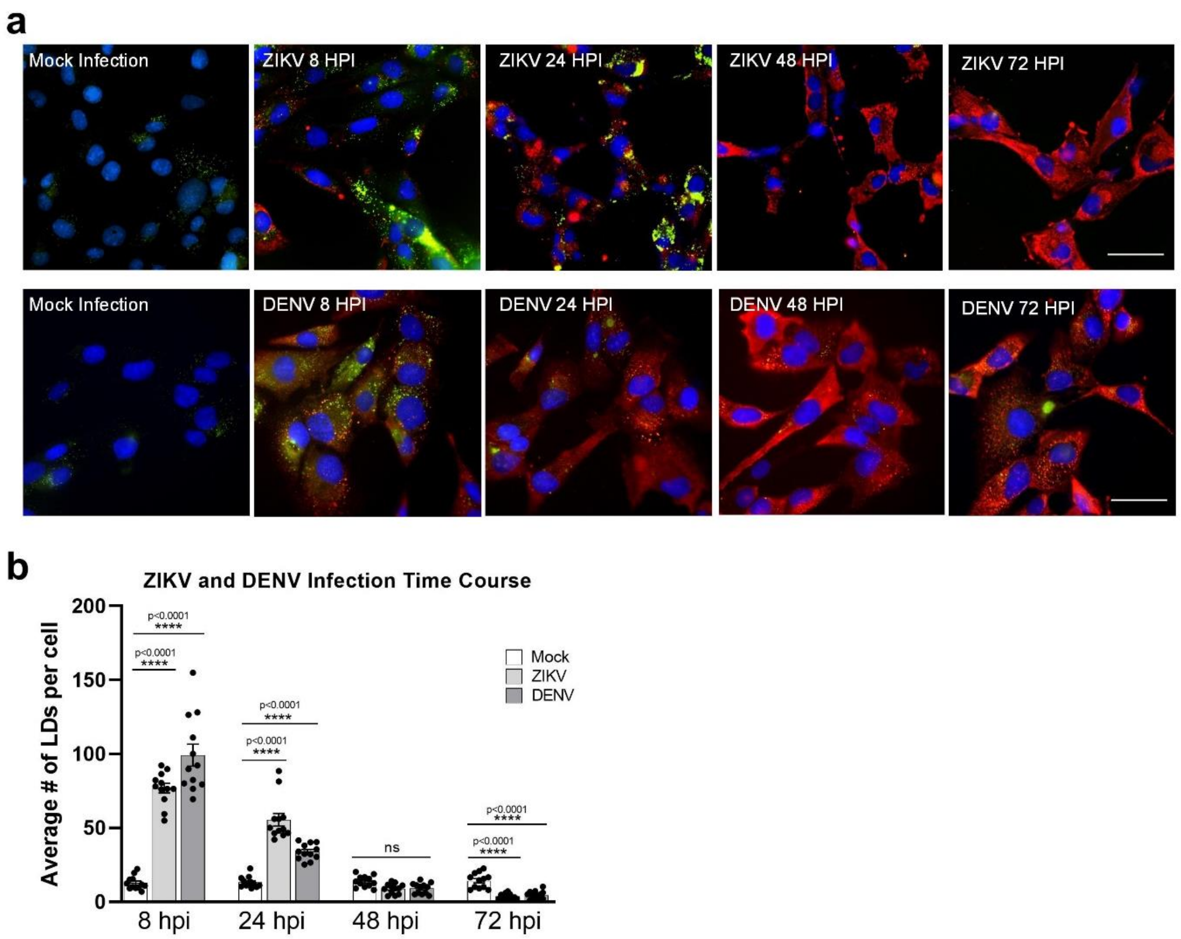

Supplementary Figure 9. LDs are induced upon initial ZIKV infection but are downregulated by 48 hrs post infection. (a) Primary immortalised astrocyte cells were infected with ZIKV strain MR766 or DENV at MOI 5 for up to 72 hrs post infection. Cells were stained with Bodipy (493/503) to visualise LDs (green) and DAPI to visualise the cell nuclei (blue), ZIKV and DENV RNA was detected using an anti-3G1.1 and 2G4 dsRNA antibody (red). **(b)** The average number of LDs was analysed per cell with ImageJ analysis software. Error bars, mean values ± SEM, P-values were determined by unpaired two-tailed Student's t test with a Holm-Sidak correction for multiple comparisons (greater than 300 cells; n=3 biological replicates). Stimulated cells were statistically compared with their respective mock controls, ns= not significant. Source data are provided as a Source Data file.

Supplementary Table 1: Primer sequences

**Primer name**	**Sequence**
RPLOPO-FP	5’-AGA TGC AGC AGA TCC GCA T-3’
RPLPO-RP	5’-GGA TGG CCT TGC GCA-3’
IFN-β-FP	5’-AGA AAG GAC GAA CAT TGG GAA A-3’
IFN-β-RP	5’-TAG CAG AGC CCT TTT TGA TAA TGT AA-3’
IFN-λ -FP	5’-GAA GAG TCA CTC AAG CTG AAA AAC-3’
IFN-λ-RP	5’-AGA AGC CTC AGG TCC CAA TTC-3’
Viperin-FP	5’GTG AGC AAT GGA AGC CTG ATC-3’
Viperin-RP	5’-GCT GTC ACA GGA GAT AGC GAG AA-3’
ZIKV-FP	5’CAG CTG GCA TCA TGA AGA AGA AYC-3’
ZIKV-RP	5’CAC YTG TCC CAT CTT YTT CTC C-3’
HSV-1-FP	5’-TCG GCG TGG AAG AAA CGA GAG A-3’
HSV-1-RP	5’-CGA ACG CAC CCA AAT CGA CA-3’
