# Supplementary material for: Intracellular lipid droplet accumulation occurs early following viral infection and is required for an efficient interferon response
Source: Nat Commun. 2021 Jul 14;12:4303. doi: 10.1038/s41467-021-24632-5 (PMC8280141; doi:10.1038/s41467-021-24632-5)
Supplement: Supplementary file 3 — Reporting Summary [file 41467_2021_24632_MOESM3_ESM.pdf]

nature research

Corresponding author(s): Assoc. Prof. Karla Helbig
Last updated by author(s): May 24th, 2021

Reporting Summary

Nature Research wishes to improve the reproducibility of the work that we publish. This form provides structure for consistency and transparency in reporting. For further information on Nature Research policies, see Authors & Referees and the Editorial Policy Checklist .

Statistics

For all statistical analyses, confirm that the following items are present in the figure legend, table legend, main text, or Methods section.

n/a	Confirmed	
☐	☒	The exact sample size (*n*) for each experimental group/condition, given as a discrete number and unit of measurement
☐	☒	A statement on whether measurements were taken from distinct samples or whether the same sample was measured repeatedly
☐	☒	The statistical test(s) used AND whether they are one- or two-sided<br>*Only common tests should be described solely by name; describe more complex techniques in the Methods section.*
☒	☐	A description of all covariates tested
☒	☐	A description of any assumptions or corrections, such as tests of normality and adjustment for multiple comparisons
☐	☒	A full description of the statistical parameters including central tendency (e.g. means) or other basic estimates (e.g. regression coefficient) AND variation (e.g. standard deviation) or associated estimates of uncertainty (e.g. confidence intervals)
☐	☒	For null hypothesis testing, the test statistic (e.g. *F*, *t*, *r*) with confidence intervals, effect sizes, degrees of freedom and *P* value noted<br>*Give P values as exact values whenever suitable.*
☒	☐	For Bayesian analysis, information on the choice of priors and Markov chain Monte Carlo settings
☒	☐	For hierarchical and complex designs, identification of the appropriate level for tests and full reporting of outcomes
☒	☐	Estimates of effect sizes (e.g. Cohen's *d*, Pearson's *r*), indicating how they were calculated

Our web collection on statistics for biologists contains articles on many of the points above.

Software and code

Policy information about availability of computer code

Data collection	Data for microscopy experiments (Figures 1A,B,C & D Figure 2A & D, Figure 3A, Figure 4A, Figure 6A & C, Figure 7 A, C &G, Supp 1 A & B, supp 2 A & B, Supp 4A, Supp 6 A & B, Supp 7 A and Supp 9A) were collected through NIS Elements software AR v.3.22.<br>Data for RT-PCR experiments (Figure 3A, Figure 4D & E, Figure 5A, B, C & D, Figure 8A, B C & D and Supp 8A) were collected on a Biorad CFX Connect Real-Time Detection System machine.<br>Data for ELISA experiments (Figure 4F & G, Figure 5E) was collected using a CLARIOstar plate reader (BMG labtech) with MARS Data analysis software (Version 1.20).
Data analysis	For analysis of microscopy experiments (Figures 1A,B,C & D Figure 2A & D, Figure 3A, Figure 4A, Figure 6A & C, Figure 7 A, C &G, Supp 1 A & B, supp 2 A & B, Supp 4A, Supp 6 A & B, Supp 7 A and Supp 9A) ImageJ analysis softwares (Version 2.1) basic particle analysis tool was used to analyse the average size and number of lipid droplets.<br>All data was analysed using Prism 8 analysis software as described in the manuscript to determine statistical significance.

For manuscripts utilizing custom algorithms or software that are central to the research but not yet described in published literature, software must be made available to editors/reviewers. We strongly encourage code deposition in a community repository (e.g. GitHub). See the Nature Research guidelines for submitting code & software for further information.

Data

Policy information about availability of data

All manuscripts must include a data availability statement. This statement should provide the following information, where applicable:

- Accession codes, unique identifiers, or web links for publicly available datasets
- A list of figures that have associated raw data
- A description of any restrictions on data availability

The data that support this study are available within the article and its Supplementary Information files or available from the authors upon request. Source data are provided with this paper.

Field-specific reporting

Please select the one below that is the best fit for your research. If you are not sure, read the appropriate sections before making your selection.

- [ ] Life sciences
- [x] Behavioural & social sciences
- [ ] Ecological, evolutionary & environmental sciences

For a reference copy of the document with all sections, see nature.com/documents/nr-reporting-summary-flat.pdf

Behavioural & social sciences study design

All studies must disclose on these points even when the disclosure is negative.

Study description	*Briefly describe the study type including whether data are quantitative, qualitative, or mixed-methods (e.g. qualitative cross-sectional, quantitative experimental, mixed-methods case study).*
Research sample	*State the research sample (e.g. Harvard university undergraduates, villagers in rural India) and provide relevant demographic information (e.g. age, sex) and indicate whether the sample is representative. Provide a rationale for the study sample chosen. For studies involving existing datasets, please describe the dataset and source.*
Sampling strategy	*Describe the sampling procedure (e.g. random, snowball, stratified, convenience). Describe the statistical methods that were used to predetermine sample size OR if no sample-size calculation was performed, describe how sample sizes were chosen and provide a rationale for why these sample sizes are sufficient. For qualitative data, please indicate whether data saturation was considered, and what criteria were used to decide that no further sampling was needed.*
Data collection	*Provide details about the data collection procedure, including the instruments or devices used to record the data (e.g. pen and paper, computer, eye tracker, video or audio equipment) whether anyone was present besides the participant(s) and the researcher, and whether the researcher was blind to experimental condition and/or the study hypothesis during data collection.*
Timing	*Indicate the start and stop dates of data collection. If there is a gap between collection periods, state the dates for each sample cohort.*
Data exclusions	*If no data were excluded from the analyses, state so OR if data were excluded, provide the exact number of exclusions and the rationale behind them, indicating whether exclusion criteria were pre-established.*
Non-participation	*State how many participants dropped out/declined participation and the reason(s) given OR provide response rate OR state that no participants dropped out/declined participation.*
Randomization	*If participants were not allocated into experimental groups, state so OR describe how participants were allocated to groups, and if allocation was not random, describe how covariates were controlled.*

Reporting for specific materials, systems and methods

We require information from authors about some types of materials, experimental systems and methods used in many studies. Here, indicate whether each material, system or method listed is relevant to your study. If you are not sure if a list item applies to your research, read the appropriate section before selecting a response.

Materials & experimental systems

n/a	Involved in the study	
☐	☒	Antibodies
☐	☒	Eukaryotic cell lines
☒	☐	Palaeontology
☐	☒	Animals and other organisms
☒	☐	Human research participants
☒	☐	Clinical data

Methods

n/a	Involved in the study	
☒	☐	ChIP-seq
☒	☐	Flow cytometry
☒	☐	MRI-based neuroimaging

Antibodies

Antibodies used	1. Mouse anti-Influenza nucleoprotein (NP) Ab (Cat#11675-MM03T, Clone #7B4G10G8, Lot #unknown Sino Biological, Beijing, China); 2. Mouse anti-3G1.1 and 2G4 dsRNA Ab (Gifted from Roy Hall, University of Queensland, these antibodies are not commercially available and have been validated by the scientists O'Brien et al PLoS Negl Trop Dis 2015); 3. Rabbit anti-HSV-1 Ab (Cat#ab9533, Lot#GR3194089-2, Abcam Cambridge, UK); 4. Goat Anti-Mouse IgG Alexa Fluor® 555 (Cat#A-21422, Lot#2214478 , Life Technologies); 5. Goat anti-Rabbit IgG (H+L) Alexa Fluor® 555 (Cat#A32732, Lot#1903133, Life Technologies).6.MAR1 antibody was a kind gift from Professor Paul Hertzog, Hudson Institute, Melbourne (This antibody is not commercially available but has been extensively validated and is published Sheehan, K. C. F. et al. J. Interferon Cytokine Res 2006).
Validation	1. Mouse anti-Influenza nucleoprotein (NP) Ab (Cat#11675-MM03T, Clone #7B4G10G8, Lot #unknown Sino Biological, Beijing,

China). Validated for IF in 'Nanostructured glycan architecture is important in the inhibition of influenza A virus infection' (PubMed ID: 27775724) and in 'Inactivated H7 influenza virus vaccines protect mice despite low levels of neutralizing antibodies' (PubMed ID: 28768855). This antibody was used at a 1:1000 dilution, at room temperature for 1 hour.
2. Mouse anti-3G1.1 and 2G4 dsRNA Ab (Gifted from Roy Hall, University of Queensland, these antibodies are not commercially available and have been validated by the scientists O'Brien et al PLoS Negl Trop Dis 2015). This antibody was used neat, at room temperature for 1 hour.
3. Rabbit anti-HSV-1 Ab (Cat#ab9533, Lot#GR3194089-2, Abcam Cambridge, UK). Validated for IF in 'NLRP3, NLRP12, and IFI16 Inflammasomes Induction and Caspase-1 Activation Triggered by Virulent HSV-1 Strains Are Associated With Severe Corneal Inflammatory Herpetic Disease.' (PubMed: 31367214) and in 'MiR-155-5p modulates HSV-1 replication via the epigenetic regulation of SRSF2 gene expression.' (PubMed: 30950329). This antibody was used at a 1:200 dilution, at room temperature for 1 hour.
6. MAR1 antibody was manufactured by Paul hertzog, Hudson Institute, Melbourne. The antibody was published in Sheehan et al., J Interferon Cytokine Res (2006), and was originally validated for binding and blocking murine IFNAR1 in vitro and in vivo.

Eukaryotic cell lines

Policy information about cell lines

Cell line source(s)

-Primary Immortalized Human Astrocyte cells were purchased from abmgood, Canada.
-Primary Murine Astrocyte cells were extracted by us as described in the manuscript (Ethics approval from La Trobe University; AEC18-05).
-Primary Murine Embryonic Fibroblasts (MEFs) were gifted from Prof. Michael Beard, University of Adelaide and have been described previously (https://www.nature.com/articles/s41598-017-04138-1).
-THP-1 monocytic leukaemia cells were a kind gift from Assoc. Prof. Ivan Poon at La Trobe University, Melbourne; these cells were originally obtained from ATCC
-HeLa human epithelial cells which a cervical carcinoma cell line were laboratory stock gifted from the Institute of Medical and Veterinary Science, Adelaide. and were originally obtained from ATCC.
-Vero cells are kidney epithelial cells extracted from an African green monkey and were a kind gift from Prof. Jason McKenzie at the Peter Doherty Institute for Infection and Immunity, Melbourne and were originally obtained from ATCC.
- MCF7 cells were a kind gift from Belinda Parker (Peter Mac Institute, Melbourne), and were originally obtained from ATCC.

-Huh-7 cells were a kind gift from Professor Michael Beard and were originally obtained from ATCC.

Authentication

None of the cell lines used were authenticated

Mycoplasma contamination

All cell lines are regularly tested for mycoplasma contamination using the LookOut® Mycoplasma PCR Detection Kit (Sigma-Aldrich) and no cell lines used in this study were positive for contaimination.

Commonly misidentified lines
(See ICLAC register)

No commonly misidentified cell lines were used in this study

Animals and other organisms

Policy information about studies involving animals; ARRIVE guidelines recommended for reporting animal research

Laboratory animals

C57BL/6 mice were used for in vivo influenza studies and harvesting of primary astrocytes and BALB/c mice were used for dengue virus infections. C57BL/6 female and male mice were 6-10 week old for influenza infections. Female C57BL/6 3-12 month old pregnant mice were used for fetal harvests. BLAB/c female and male mice were 1 day in age.

Wild animals

This study did not involve wild animals.

Field-collected samples

This study did not involve samples collected from the field.

Ethics oversight

All experiments were done in accordance with the Institutional Animal Care and Use Committee guidelines of the University of Melbourne (Murine tissue samples; Figure 1D and Supp 1B), La Trobe University (Primary murine astrocytes; Figure 2B & C) or the University of Adelaide (Primary murine embryonic fibroblasts; Figure 2B & C). C57BL/6 mice were bred in-house and housed under specific pathogen–free conditions in the animal facility at the Peter Doherty Institute of Infection and Immunity, University of Melbourne, Melbourne, Australia. Animal experiments were performed in accordance with the University of Melbourne's Animal Welfare Committee (1714189) and Institutional Biosafety Committee approval NLRD (2018/023)."

"BALB/c mice were bred in-house and housed under specific pathogen-free conditions in the animal facility at Flinders University, Adelaide, Australia. Animal procedures were performed in accordance with Flinders University Animal Welfare Committee approval number 935-17 and Institutional Biosafety Committee approval NLRD 2013-24."

'The establishment of astrocytic cultures from the brains of C57BL/6 mice (post-natal day 1.5) was performed as described previously 61. C57BL/6 mice were bred in-house and housed under specific pathogen-free conditions in the animal facility at La Trobe University; mice were maintained under a 12-h light/dark cycle at an ambient temperature of 20–23 °C and relative humidity of 40–60% and with ample food and water. Experiments were carried out under the approval of the La Trobe University Animal Ethics Committee (AEC 18-05) in accordance with Guidelines for Ethical Conduct in the Care and Use of Animals'

Note that full information on the approval of the study protocol must also be provided in the manuscript.
